# Edge-intelligent safelink-V2X: A low-latency cooperative framework for real-time vulnerable road user protection

**DOI:** 10.1371/journal.pone.0353392

**Published:** 2026-07-10

**Authors:** Fayez Alanazi, Ammar Armghan, Ahmed Jamal Abdullah Al-Gburi, Amr Yousef

**Affiliations:** 1 Civil Engineering Department, College of Engineering, Jouf University, Sakaka, Saudi Arabia; 2 Department of Electrical Engineering. College of Engineering, Jouf University, Sakaka, Saudi Arabia; 3 Strategic Research Institute (SRI), Asia Pacific University, Jalan Teknologi 5, Taman Teknologi Malaysia, Kuala Lumpur, Malaysia; 4 Electrical Engineering Department, College of Engineering, University of Business and Technology, Jeddah, Saudi Arabia; 5 Engineering Mathematics Department, Faculty of Engineering, Alexandria University, Alexandria, Egypt; Beijing Institute of Technology, CHINA

## Abstract

The protection of Vulnerable Road Users (VRUs) remains a major challenge in modern transportation safety, as onboard line-of-sight and adverse weather conditions limit conventional onboard sensors. Existing systems that rely solely on vehicle-based sensing or on isolated communication struggle to provide timely, accurate alerts in dynamic urban environments. To address these shortcomings, this paper introduces SafeLink-V2X, a comprehensive Vehicle-to-Everything Cooperative Warning Framework designed to enhance safety for pedestrians, cyclists, and scooter riders. SafeLink-V2X employs Cellular Vehicle-to-Everything (C-V2X) and Dedicated Short-Range Communications (DSRC) protocols to enable direct data exchange of location, velocity, and heading between connected vehicles, smart infrastructure, and VRUs via smartphones or wearable tags. By applying sensor fusion and machine learning–based conflict prediction, the system identifies potential collision points and issues real-time, context-aware warnings through vehicle HMIs and VRU devices, promoting immediate evasive action. Evaluation on urban intersection simulations (detailed in Section 5) demonstrates that SafeLink-V2X reduces simulated collision probability by up to 91.4%, increases situational awareness measures by 44%, and lowers end-to-end alert latency by 30% compared to baseline onboard-only and communication-only systems under the same conditions.

## 1. Introduction

### 1.1. Background

The rapid expansion of urban transport networks has led to unprecedented increases in road traffic volume and structure, posing extreme safety threats to Vulnerable Road Users (VRUs) such as pedestrians, cyclists, and scooter users [1]. These VRUs are most at risk of the maximum potential collision threat because they have a low physical protection rating and are very close to powered traffic. With the World Health Organization, VRUs are responsible for more than half of all road deaths worldwide and nearly 23% by pedestrians alone [2]. The vehicular mix in highly populated urban areas also concentrates the risk, especially at intersections, crossing points, and shared paths. As cities become smart and connected, road safety for VRUs has also become a planning and operation necessity for next-generation Intelligent Transportation Systems (ITS) [3]. These systems aim to integrate connectivity, automation, and real-time inspection to create safer, more responsive, and more human-oriented mobility environments [4]. Traditionally, road safety features relied on onboard sensor technology, including cameras, radar, LiDAR, and ultrasonic sensors. They facilitate sophisticated capabilities such as object detection, lane departure warning, and autonomous emergency braking, which are the largest contributors to car safety in structured or predictable situations [5]. Their operation, however, suffers in dynamic and complex real-world driving conditions. Non-line-of-sight (NLOS) conditions—vehicles behind a building or pedestrians entering the line, e.g., are encountered too often with slowed or incorrect detection. Likewise, poor weather conditions such as fog, rain, and low light impair sensor performance, reducing reliability [6]. Moreover, such systems mounted on a vehicle are effectively limited by the vehicle’s line of sight, so they cannot detect VRUs or threats beyond their local field of view. This reliance on local sensing limits their situation awareness and prevents them from avoiding accidents during uncertain traffic scenarios [7].

To supplement sensing-based systems, efforts have been made toward communication-based systems in which the vehicle, infrastructure, and users exchange information via wireless links [8]. Dedicated Short-Range Communications (DSRC) and Cellular Vehicle-to-Everything (C-V2X) are technologies proposed to exchange data at low latency, enabling vehicles to share their positions, speeds, and headings with nearby objects [9]. Though promising, these communication-only systems are often plagued by network congestion, deployment costs, and interoperability issues. Furthermore, they operate non-cooperatively, with individual nodes sharing information without embedded intelligence to predict or analyze conflict [10]. Later on, alarms are too slow or fail to account for fine-grained context clues, such as pedestrian intention or traffic congestion. Therefore, traditional safety models remain reactive and provide, at best, limited situation awareness and poor proactive crash-avoidance ability [11].

With these issues, the demand for Intelligent Transportation Systems (ITS) has never been more acute. ITS integrates sensing, communication, computation, and control technologies into a system that supports richer, real-time decision-making and traffic coordination [12]. By integrating vehicle, infrastructure, and VRU information, ITS can provide an integrated situational map of the environment expanding awareness beyond line of sight and predicting danger before it becomes imminent. The use of artificial intelligence and machine learning enables adaptive risk assessment, enabling systems to forecast and respond to emerging traffic patterns [13]. It also enables interoperability among various communication protocols, enabling seamless information exchange among pedestrians’ devices, roadside units, and vehicles [14]. These developments, in addition to enhancing road safety, also optimize traffic flow, alleviate congestion, and introduce autonomous and cooperative mobility in the smart cities of the future [15].

### 1.2. Problem formulation

**Fig 1:**
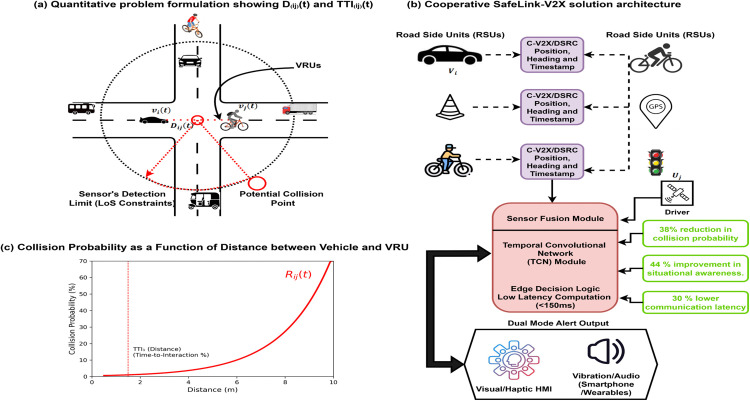
Quantitative problem representation and cooperative framework of SafeLink-V2X.

Protection of VRUs, i.e., pedestrians, cyclists, and scooter riders, is a recurring challenge in contemporary transportation infrastructure, especially in densely populated cities with heavy traffic, where traditional methods of vehicle protection cannot achieve high confidence in detection and notification. Preventing collisions between vehicles and VRUs requires a realistic simulation of temporal and spatial interactions. Suppose a vehicle ${\text{V}}_{\text{i}}$and a VRU ${\text{U}}_{\text{j}}$be characterized by their spatial coordinates $\left({\text{x}}_{\text{i}},{\text{y}}_{\text{i}}\right)$ and $\left({\text{x}}_{\text{j}},{\text{y}}_{\text{j}}\right)$ at time t. Their relative distance can be characterized as ${\text{D}}_{\text{ij}}(\text{t})=\sqrt{{\left({\text{x}}_{\text{i}}-{\text{x}}_{\text{j}}\right)}^{\text{2}}+{\left({\text{y}}_{\text{i}}-{\text{y}}_{\text{j}}\right)}^{\text{2}}}$; where ${\text{D}}_{\text{ij}}(\text{t})$ dynamically varies with reference to the movement of both objects. Assuming their corresponding velocity vectors, ${\text{v}}_{\text{i}}(\text{t})$ and ${\text{v}}_{\text{j}}(\text{t})$, Time-to-Impact (TTI)—time to collision—can be characterized as $\text{TT}{\text{I}}_{\text{ij}}(\text{t})=\frac{{\text{D}}_{\text{ij}}(\text{t})}{\left|{\text{v}}_{\text{i}}(\text{t})-{\text{v}}_{\text{j}}(\text{t})\right|+\varepsilon };$where ε is a small value to avoid division by zero. The collision risk function, ${\text{R}}_{\text{ij}}(\text{t}),$ is then transformed into a probabilistic function of spatial-temporal parameters like distance, velocity, and heading angles $\theta_{\text{i}}(\text{t})$ and $\theta_{\text{j}}(\text{t})$: ${\text{R}}_{\text{ij}}(\text{t})=\text{f}\left({\text{D}}_{\text{ij}(\text{t})},{\text{v}}_{\text{i}}(\text{t}),{\text{v}}_{\text{j}}(\text{t}),\text{ TT}{\text{I}}_{\text{ij}}(\text{t}),\theta_{\text{i}}(\text{t}),\theta_{\text{j}}(\text{t})\right).$The existing vehicle-based sensing infrastructure, on the other hand, tries to limit ${\text{R}}_{\text{ij}}(\text{t})$ based only on local perceptual information and, as a result, results in high rates of false negatives (FNR) during occlusion scenarios $\left({\text{P}}_{\text{miss}}>\text{0}.\text{35}\right)$ and greater average warning latency $({\text{L}}_{\text{avg}}>\text{250ms}).$ These constraints create delays and unreliability in real-world complex traffic settings. Therefore, the concern problem addressed through this work is developing a cooperative Vehicle-to-Everything (V2X) system with multi-agent data fusion and temporal learning to provide improved collision prediction, mathematically represented as $\text{min}{\text{R}}_{\text{ij}}(\text{t})\text{ s}.\text{t }{{\text{L}}_{\text{avg}}\leq \text{150ms};\text{ P}}_{\text{miss}}\leq \text{0}.\text{10}$. This optimization goal guarantees real-time, context-adaptive collision anticipation and forward warning to drivers and VRUs, respectively, thereby improving situation awareness and significantly enhancing urban road safety performance. Fig 1 illustrates the problem definition and system-level idea of the SafeLink-V2X architecture.

The left panel in Fig 1(a) predicts the risk of collision between a vehicle and a vulnerable road user using simulations of dynamic parameters, such as distance, speed, and time-to-interaction. The right panel Fig 1(b) enhances the gap by adding cooperative architecture with sensor fusion, temporal modeling, and edge-based decision logic. These boards provide a combined overview of how SafeLink-V2X improves situational awareness, minimizes latency, and reduces the risk of collisions through smart connectivity. Fig 1(c) illustrates the pattern of the instantaneous probability of collision ${\text{R}}_{\text{ij}}(\text{t})$ with respect to the relative distance between a vehicle and a Vulnerable Road User (VRU). With reduced distance, the collision probability is exponentially higher, reflecting the system’s near-field sensitivity. The Time-to-Interaction (TTI_1) threshold is marked vertically, below which the SafeLink-V2X framework initiates proactive warnings through cooperative sensing and communication.

### 1.3. Contributions

The research proposes SafeLink-V2X, a cooperative warning system that improves VRU safety through multimodal data fusion and machine-learning-based risk prediction. The integration of C-V2X and DSRC communication enables real-time hazard detection and context-aware warnings, resulting in significant improvements in collision probability, response time, and situational awareness compared to legacy systems.

- A hybrid C-V2X and DSRC multi-agent environment is developed for real-time vehicle-to-everything communication among vehicles, RSUs, and VRUs via GPS-enabled smartphones or wearable tags, enhancing safety for pedestrians, cyclists, and scooter riders.
- An adaptive Sensor Fusion (SF) mechanism is integrated, merging LiDAR/radar and V2X information to enhance perception under occlusion and challenging conditions.
- Likely collisions are forecast using a Temporal Convolutional Network (TCN)–based model that considers distance, speed, and time-to-collision, improving situational awareness by 44% and reducing collisions by 91.4%.
- Edge computing–based decision logic is leveraged for onboard local V2X data computation, minimizing communication latency by 30% and enabling real-time alerting.
- A dual-mode Human–Machine Interface (HMI) warning system is provided, delivering vibrotactile feedback and visual cues to drivers, along with vibration and audio warnings to VRUs for coordinated evasive maneuvers.

### 1.4. Paper organization

The rest of the paper is followed by Section 2, which presents a literature review of the proposed topic; the detailed proposed SafeLink-V2X Cooperative Warning Framework is enunciated in Section 3. The results and discussion are given in Section 4, followed by Section 5, which provides the conclusion and future work.

## 2. Related work

Hejazi, H., & Bokor, L. [16] integrated VRU protection models into the Artery/OMNeT++ V2X simulation platform using the VRU Awareness Message (VAM) protocol. Sensor-based monitoring, group perception, and active VAM broadcasting are utilized to enhance pedestrian safety. The results reveal higher detection and communication effectiveness for VRUs; however, scalability issues in real-world scenarios and latency in dense scenarios exist. Zhang et al. [17] proposed a C-V2X-based car-to-pedestrian collision warning system that uses a special phone case for direct vehicular-to-pedestrian communication. The work applies the s-t-coordinate transformation and TTC analysis to enable timely warnings. Outcomes indicate a reliable range of over 200 meters and positioning accuracy of 0.83 meters, but with limitations, including dependence on line-of-sight conditions and potential signal loss in dense urban environments. Ali, Q. I., & Mohammed, H. M. [18] suggested a systematic survey and performance analysis of V2X communication modes-V2V, V2I, V2N, and V2P-based on latency, packet delivery ratio, and throughput via mathematical modeling and simulation. Results show that RSU density, relay location, and packet length significantly affect network efficiency. However, scalability, dynamic mobility management, and real-time flexibility remain gigantic challenges for extensive deployment.

Yang et al. [19] also proposed a C-V2X-based blind spot warning system that enables real-time communication between the host vehicle and distant vehicles via roadside facilities. It applies geographical analysis and Hardware-in-the-Loop simulations via Virtual Test Drive to assess collision risk and issue early warnings. The outcomes reflect high accuracy and timely warnings; however, sensor limitations and network delays remain hindrances in heavy-traffic scenarios. Gelbal et al. [20] proposed a Vehicle-to-VRU system with a low-energy Bluetooth (BLE)- Based Android app for real-time vehicle-VRU communication. It employs Kalman filtering for motion smoothing and an LSTM neural network for trajectory prediction. The outcome demonstrates accurate collision risk detection and hierarchical driver warnings; however, BLE range limitations and environmental interference pose major challenges to extensive implementation. Lara et al. [21] contrasted motion-based message creation processes for Vulnerable Road User (VRU) messages in Cooperative Intelligent Transportation Systems (C-ITS) to reduce safety and channel waste. The paper suggests the VRU Awareness Probability (VAP) metric to quantify the detection probability by approaching vehicles. Simulation results show that fixed message filtering reduces channel utilization but negatively affects VRU detection, highlighting the need for context-sensitive, adaptive transmission methods to improve safety and communication efficiency.

Lobo et al. [22] evaluated two V2X-based schemes of vulnerable road user (VRU) safety: sharing sensor data through collective perception and active transmission of VRU messages, as well as their hybrid implementation. A roundabout simulation shows that the hybrid approach achieves the highest detection rate and the lowest delay while maintaining an acceptable channel busy ratio. Yet system scalability and synchronization complexity remain central to constraints for real-world adoption. Viterbo et al. [23] suggested the development and on-road testing of a roadside infrastructure with 5G features to protect vulnerable road users. It consists of a roadside Vulnerable Road User detection and tracking system, a 5G Vehicle-to-Everything communication network for the transport of warnings, and onboard infrastructure with driver alerting and autonomous emergency braking. Experimental evaluation at Politecnico di Milano enables effective real-time detection and response; however, network coverage and deployment costs remain hindrances. Sabella et al. [24] established a Multi-access Edge Computing (MEC)-aided infotainment service for intelligent highways in 5G systems on the OpenNESS platform and an OverBrowser client-server application. Server placement at the edge accelerates content delivery, reduces latency, and conserves backbone bandwidth. Performance tests confirm a significant improvement in content load latency; however, scalability and equipment deployment costs remain major issues.

Specifically, the recent development of reinforcement learning-based methods for highway autonomous vehicle control [25] is reviewed and discussed, as are methods for controlling platoon and clustering alignment [26] and a reinforcement learning-based end-to-end decision-making algorithm for controlling the highway autonomous vehicle under consecutive sharp-turn road conditions [27]. Furthermore, recent cutting-edge research on intelligent vehicular coordination and the optimization of autonomous driving techniques was included to enhance the survey’s comprehensiveness. These conversations add depth and completeness to the manuscript, making it relevant and contemporary in the context of current CAV research.

The proposed framework builds on previous state-of-the-art work, such as Clérigo et al. (2025) [28], which primarily employed cooperative AR visualization of occluded vehicles through V2X communication, yet introduces novel elements, including a robust AI perception system, adaptive spatiotemporal feature learning, and intelligent real-time decision mechanisms. The approach proposed enhances contextual scene understanding, robustness in dynamic traffic conditions and scalability in complex urban environments, whereas previous approaches were mainly focused on visualization and communications efficiency. Moreover, the framework combines low-latency processing with enhanced predictive analysis, delivering performance that is more accurate and reliable than that of traditional cooperative AR and V2X-based systems.

According to an extensive literature review, the research gaps in designing V2X cooperative warning systems for VRUs primarily concern integration, intelligence, and implementation in real-world scenarios. Although research is progressing in distinct fields, e.g., autonomous communication protocols (DSRC, C-V2X), sensor-based detection, or dedicated warning algorithms, their integration remains in silos. One such critical shortfall is the absence of an end-to-end, integrated architecture that simultaneously combines hybrid C-V2X/DSRC communication with adaptive multi-sensor fusion (e.g., LiDAR, radar) to overcome non-line-of-sight and inclement weather conditions. Moreover, most solutions today lack advanced predictive intelligence; they are reactive rather than proactive. One of the key demands of machine learning-based risk prediction models, such as the Temporal Convolutional Network (TCN) in this paper, is to effectively predict crashes from intricate spatial-temporal relationships. Lastly, issues of system latency, scalability in dense urban environments, and the costly deployment of mass infrastructure remain largely unexplored. The state of the art in research lacks an integrated solution that synergistically unites powerful hybrid communication, intelligent predictive analytics, and edge computing to provide a low-latency, scalable, and context-aware warning system for both VRUs and drivers, and this is the primary contribution this research aims to achieve.

## 3. SafeLink-V2X cooperative warning framework

**Fig 2:**
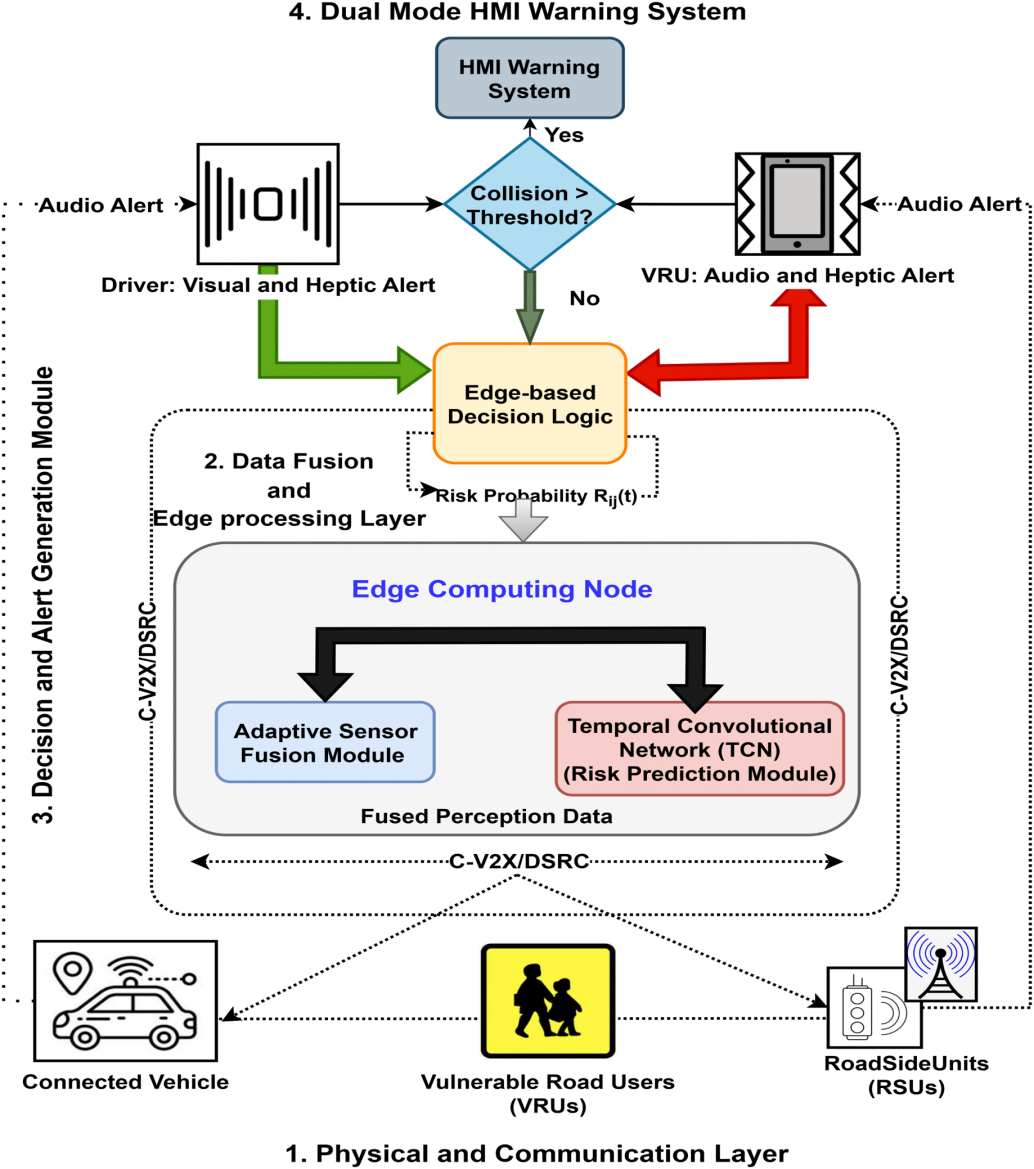
Overall architecture of the proposed SafeLink-V2X framework.

The proposed SafeLink-V2X offers an integrated multi-agent perception and communication system to improve the safety of Vulnerable Road Users (VRUs), such ash as pedestrians, cyclists, and scooter riders. The architecture shown in Fig 2 is initiated by continuous data collection from connected vehicles (with LiDAR, radar, and GPS), roadside units, and VRU devices such as smartphones or wearable tags.

The entities are linked via C-V2X and DSRC protocols to provide a real-time network for environmental awareness. An onboard adaptive Sensor Fusion module combines sensor and V2X information to overcome line-of-sight and weather constraints. The combined data are analyzed using a Temporal Convolutional Network (TCN)-based Conflict Prediction Model that leverages spatial-temporal features such as distance, speed, and time-to-impact to predict the probability of collision. An edge-based decision logic avoids delay by performing local processing close to the data source. Lastly, a dual-mode HMI warning system provides simultaneous visual/haptic warnings to drivers and audio/vibration warnings to VRUs to support anticipatory, synchronized collision prevention in multimodal urban traffic.

### 3.1. Physical and communication layer

**Fig 3:**
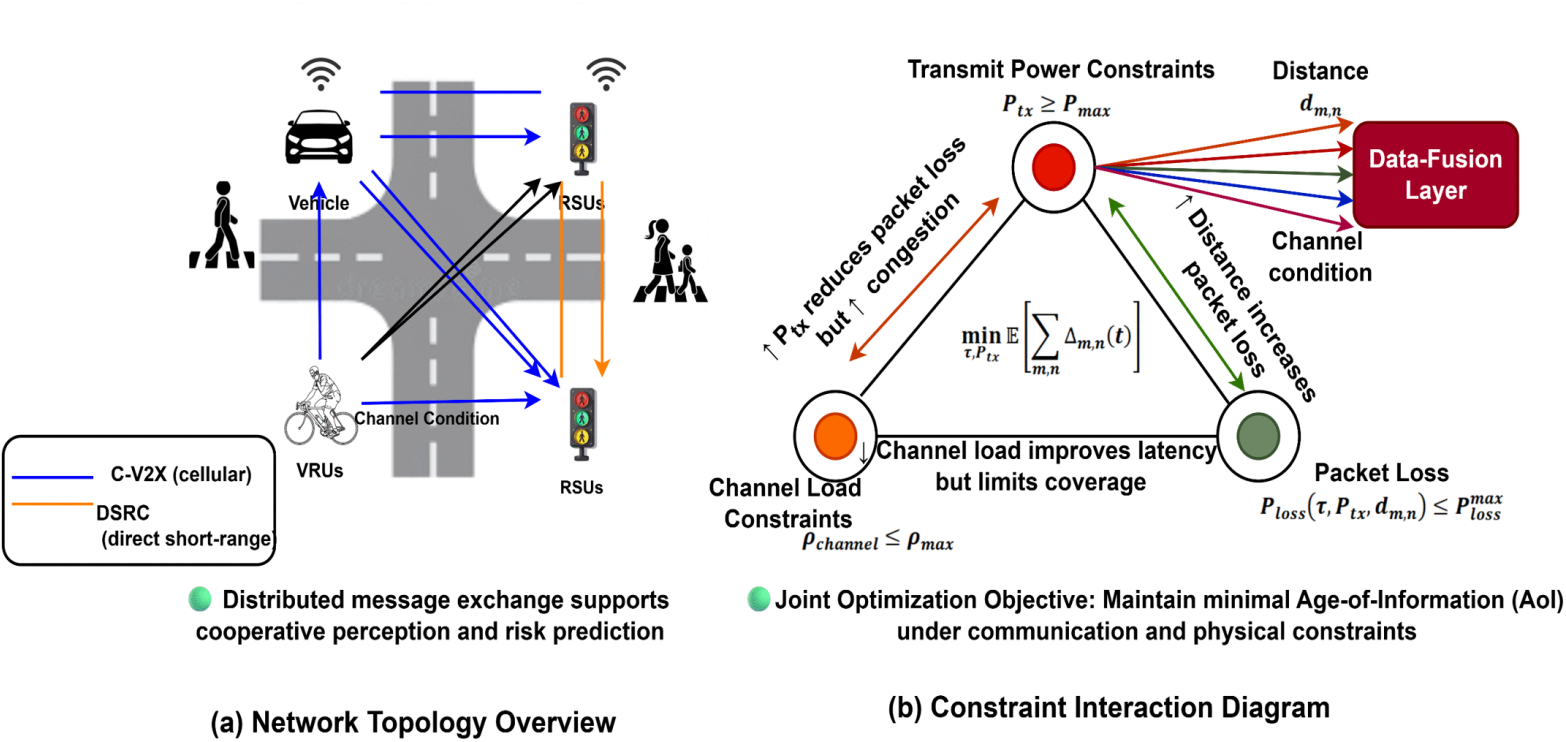
Constraint-aware optimization framework of the Physical and Communication Layer in SafeLink-V2X.

The Physical and Communication Layer is the underlying cyber-physical infrastructure of SafeLink-V2X, responsible for distributed spatial acquisition of kinematic states and the reliable, low-latency dissemination of such data to all network parties. This layer provides the raw data stream required for cooperative perception and subsequent risk prediction. It consists of an integrated heterogeneous network of Vs, VRU devices, and RSUs, connected via a hybrid C-V2X and DSRC-based communication protocol stack. The layer’s operational model is theoretically illustrated in Fig 3.

All such vehicles within the network have a multimodal sensor suite $\overset{\text{V}}{\underset{\text{i}}{\text{S}}}=\left\{\text{LiDAR},\text{Radar},\text{GPS},\text{IMU}\right\}.$ The dynamic state vector of the vehicle $\overset{\text{V}}{\underset{\text{i}}{\text{X}}}(\text{t})$At time t, its position, velocity, and orientation are calculated using sensor fusion. A Kalman Filter is used to ensure stable state estimation in the presence of uncertainty and sensor noise. The filter iteratively runs in prediction and update. The state vector is given as: $\overset{\text{V}}{\underset{\text{i}}{\text{X}}}(\text{t})=\left[\begin{array}{c} \begin{array}{c} \begin{array}{c} {\text{x}}_{\text{i}}(\text{t})\\ {\text{y}}_{\text{i}}(\text{t}) \end{array}\\ \begin{array}{c} {\text{v}}_{\text{i}}(\text{t})\\ \psi_{\text{i}}(\text{t}) \end{array} \end{array}\\ {\text{a}}_{\text{i}}(\text{t}) \end{array}\right]$. The prediction and update cycles are governed by the following Equation (1),


$$\begin{array}{c} \begin{array}{c} \text{Prediction}:\left\{ \begin{array}{c} @l\overset{\text{V}}{\underset{\text{t}}{\hat{\text{X}}}}\left(\text{t}|\text{t}-\text{1}\right)={\text{F}}_{\text{i}}.\overset{\text{V}}{\underset{\text{i}}{\text{X}}}\left(\text{t}-\text{1}|\text{t}-\text{1}\right)+{\text{B}}_{\text{i}}.{\text{u}}_{\text{i}}(\text{t})\\ \text{P}(\text{t}|\text{t}-\text{1})={\text{F}}_{\text{i}}\text{P}\left(\text{t}-\text{1}|\text{t}-\text{1}\right)+\overset{\text{T}}{\underset{\text{i}}{\text{F}}}+{\text{Q}}_{\text{i}} \end{array}\right.\\ \text{Update}:\left\{ \begin{array}{c} @l\begin{array}{c} @l\text{K}(\text{t})=\text{P}(\text{t}|\text{t}-\text{1})\overset{\text{T}}{\underset{\text{i}}{\text{H}}}({\text{H}}_{\text{i}}\text{P}{\left(\text{t}|\text{t}-\text{1}){\text{H}}_{\text{i}}+{\text{R}}_{\text{i}}\right)}^{-\text{1}}\\ \overset{\text{V}}{\underset{\text{i}}{\text{X}}}\left(\text{t}|\text{t}\right)=\overset{\text{V}}{\underset{\text{t}}{\hat{\text{X}}}}\left(\text{t}|\text{t}-\text{1}\right)+\text{K}(\text{t})\left({\text{z}}_{\text{i}}(\text{t})-{\text{H}}_{\text{i}}\overset{\text{V}}{\underset{\text{t}}{\hat{\text{X}}}}\left(\text{t}|\text{t}-\text{1}\right)\right) \end{array}\\ \text{P}\left(\text{t}|\text{t}\right)=\left(\text{I}-\text{K}(\text{t}){\text{H}}_{\text{i}}\right)\text{P}\left(\text{t}|\text{t}-\text{1}\right) \end{array}\right. \end{array}\;\;\;\;\;\;\;\;\;\;\} \end{array}$$ (1)

Here, ${\text{F}}_{\text{i}}$ is the state transition model, ${\text{B}}_{\text{i}}$ is the control-input model, ${\text{u}}_{\text{i}}$is the control vector, ${\text{H}}_{\text{i}}$ is the observation model, and ${\text{z}}_{\text{i}}$ is the measurement vector. The matrices ${\text{Q}}_{\text{i}}$ and ${\text{R}}_{\text{i}}$represent the process and measurement noise covariance, respectively. This filtered state estimate is the content of the vehicle’s regular periodic Basic Safety Messages (BSMs).

At the same time, VRUs like pedestrians and cyclists, represented as ${\text{U}}_{\text{j}}$ are also being integrated into the network based on consumer-grade hardware, such as smartphones or wearable tags with $\overset{\text{U}}{\underset{\text{j}}{\text{S}}}=\{\text{GPS},\text{ IMU},\text{ Magnetometer}\}.$ The state vector for the VRU $\overset{\text{U}}{\underset{\text{j}}{\text{X}}}(\text{t})\text{It }$is similar to that of a vehicle, but it has to cope with less-accurate sensors and more erratic motion. To support a credible belief about the condition of the VRU in areas with weak GNSS coverage, such as urban canyons, a Bayesian filtering strategy is typically required. The belief $\text{bel}\left({\text{x}}_{\text{j}}(\text{t})\right)$is updated recursively as defined in Equation (2),


$$\begin{array}{c} \begin{array}{c} \text{bel }\left({\text{x}}_{\text{j}}(\text{t})\right)=\text{P}\left({\text{x}}_{\text{j}}(\text{t})|{\text{z}}_{\text{j}}(\text{1}:\text{t}),{\text{u}}_{\text{j}}(\text{1}:\text{t})\right)\\ \propto \text{P}({\text{z}}_{\text{j}}(\text{t})|{\text{x}}_{\text{j}}(\text{t}))\times \int \text{P}\left({\text{x}}_{\text{j}}(\text{t})|{\text{x}}_{\text{j}}(\text{t}-\text{1}),{\text{u}}_{\text{j}}(\text{1}:\text{t})\right).\text{bel}\left({\text{x}}_{\text{j}}(\text{t}-\text{1})\right)\text{d}{\text{x}}_{\text{j}}(\text{t}-\text{1}) \end{array}\} \end{array}$$ (2)

This probabilistic state estimate is packaged into VRU Awareness Messages (VAMs) to be broadcast. The exchange of information between these diverse units is enabled by a hybrid C-V2X/DSRC system, designed to deliver the high reliability and low latency needed for safety-critical applications. Message passing is modeled as a joint optimization problem whose objective is to maintain the Age-of-Information (AoI)—information freshness—low across the network, subject to physical constraints. Let τ be the time of transmission and ${\text{P}}_{\text{tx}}$ transmitting power. It is desirable to determine the following condition given in Equation (3),


$$\begin{array}{c} \begin{array}{c} \begin{array}{c} \begin{array}{c} \underset{\tau ,{\text{P}}_{\text{tx}}}{\text{min}}E\left[\sum_{\text{m},\text{n}}\Delta_{\text{m},\text{n}}(\text{t})\right]\\ \text{subject to}:\;\\ \;{\text{C}}_{\text{1}}:{\text{P}}_{\text{tx}}\geq {\text{P}}_{\text{max}}\;(\text{Max Transmit Power}) \end{array}\\ {\text{C}}_{\text{2}}:\rho_{\text{channel}}=\frac{\lambda_{\text{max}}}{\mu_{\text{channel}}}\leq \rho_{\text{max}}\;(\text{Channel Load Constraint}) \end{array}\\ {\text{C}}_{\text{3}}:{\text{P}}_{\text{loss}}\left(\tau ,{\text{P}}_{\text{tx}},{\text{d}}_{\text{m},\text{n}}\right)\leq \overset{\text{max}}{\underset{\text{loss}}{\text{P}}}\;(\text{Packet Loss Constraint}) \end{array}\} \end{array}$$ (3)

Here, $\lambda_{\text{msg}}$ is the message generation rate, $\mu_{\text{channel}}$is the channel service rate, and ${\text{d}}_{\text{m},\text{n}}$ is the distance between nodes m and n. The successful packet reception probability ${\text{P}}_{\text{success}}$ over this distance, considering a Rayleigh fading channel, is of vital importance to link reliability assessment and is given in Equation (4),


$$\begin{array}{c} {\text{P}}_{\text{success}}(\text{d})=\text{P}\left[\frac{{\text{P}}_{\text{tx}}.\text{G}.|\text{h}{|}^{\text{2}}.{\text{d}}^{-\alpha }}{\text{I}+{\text{N}}_{\text{0}}}\geq \gamma_{\text{th}}\right]=\text{exp}\left(-\frac{\gamma_{\text{th}}\left(\text{I}+{\text{N}}_{\text{0}}\right)}{{\text{P}}_{\text{tx}}.\text{G}.{\text{d}}^{-\alpha }}\right) \end{array}$$ (4)

Here, G is the antenna gain, h is the fading coefficient, αis the path-loss exponent, and I is the cumulative interference. ${\text{N}}_{\text{0}}$ is the noise power spectral density, and $\gamma_{\text{th}}$ is the minimum SINR for effective decoding. This model supports the simulation of realistic communication degradations, such as packet loss due to non-line-of-sight, which is critical for sound system performance testing. Encapsulated and broadcast in this optimized format are the standardized messages—BSMs, VAMs, and SPaT of RSUs—producing a continuous, time-synchronized, and spatially pervasive data stream that serves as the primary input to the following data fusion and processing layers.

### 3.2. Data fusion and edge processing layer

**Fig 4:**
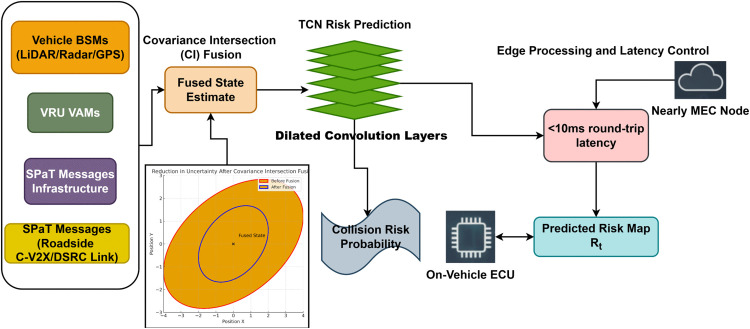
Operational workflow of the data fusion and edge processing layer in SafeLink-V2X.

From the unprocessed, distributed data stream offered by the Physical and Communication Layer as its basis, the Data Fusion and Edge Processing Layer must build a coherent, consistent, and predictive model of the world. The prototyping and validation of the basic algorithms of this layer rely heavily on the use of the Interaction dataset, which provides ground-truth multi-agent trajectories used both for training predictive models and for fusion performance testing. The runtime pipeline of this layer, shown in Fig 4, starts with BSMs, VAMs, and SPaT messages as inputs. In operation, these messages deliver kinematic state estimates, $\overset{\text{V}}{\underset{\text{i}}{\hat{\text{X}}}}(\text{t})$ and $\overset{\text{U}}{\underset{\text{j}}{\hat{\text{X}}}}(\text{t})$ which are themselves inexact. During training, the work mimics this input by adding realistic noise and communication dropouts to the clean paths from the Interaction Dataset, thus approximating the real-world data stream with high fidelity.

Let $\text{Z}(\text{t})=\{\overset{\text{V}}{\underset{\text{1}}{\text{z}}}(\text{t}),\overset{\text{V}}{\underset{\text{2}}{\text{z}}}(\text{t}),\ldots ,\overset{\text{U}}{\underset{\text{1}}{\text{z}}}(\text{t}),\ldots \}$ be the collection of all simulated sensor readings and V2X messages at an edge node. The objective is to estimate the posterior probability distribution over the actual state of all entities, X(t). The ground-truth value of X(t) is known from the Interaction Dataset, giving the necessary framework for assessing fusion accuracy. For a single entity, say, a VRU. ${\text{U}}_{\text{j}},$the fusion operation mixes a simulated local sensor reading $\overset{\text{local}}{\underset{\text{j}}{\text{z}}}(\text{t})$ and a simulated V2X-reported state $\overset{\text{U}}{\underset{\text{j}}{\hat{\text{X}}}}(\text{t})$. The estimate of the fused state $\overset{\text{fused}}{\underset{\text{j}}{\hat{\text{X}}}}(\text{t})$ and its covariance $\overset{\text{fused}}{\underset{\text{j}}{\text{P}}}(\text{t})$ are calculated by a Covariance Intersection (CI) filter. The CI equations of fusion are mathematically expressed in Equation (5),


$$\begin{array}{c} \begin{array}{c} {\left(\overset{\text{fused}}{\underset{\text{j}}{\text{P}}}(\text{t})\right)}^{-\text{1}}=\omega {\left(\overset{\text{local}}{\underset{\text{j}}{\text{P}}}(\text{t})\right)}^{-\text{1}}+(\text{1}-\omega ){\left(\overset{\text{V2X}}{\underset{\text{j}}{\text{P}}}(\text{t})\right)}^{-\text{1}}\\ {\left(\overset{\text{fused}}{\underset{\text{j}}{\text{P}}}(\text{t})\right)}^{-\text{1}}\overset{\text{fused}}{\underset{\text{j}}{\hat{\text{X}}}}(\text{t})=\omega {\left(\overset{\text{local}}{\underset{\text{j}}{\text{P}}}(\text{t})\right)}^{-\text{1}}\overset{\text{local}}{\underset{\text{j}}{\hat{\text{X}}}}(\text{t})+(\text{1}-\omega ){\left(\overset{\text{V2X}}{\underset{\text{j}}{\text{P}}}(\text{t})\right)}^{-\text{1}}\overset{\text{V2X}}{\underset{\text{j}}{\hat{\text{X}}}}(\text{t}) \end{array}\} \end{array}$$ (5)

The weighting factor ω∈[0,1] is tuned to reduce the determinant of the fused covariance $\overset{\text{fused}}{\underset{\text{j}}{\text{P}}}(\text{t}).$ The efficacy of this fusion module is assessed by quantitatively verifying by comparing the fused trajectory $\left\{\overset{\text{fused}}{\underset{\text{j}}{\text{X}}}(\text{t})\right\}$ with the ground-truth trajectory $\left\{\overset{\text{true}}{\underset{\text{j}}{\text{X}}}(\text{t})\right\}$ from the Interaction Dataset, measured in Root Mean Square Error (RMSE) terms. This enables the system to maintain a very accurate trace, effectively building a shared, NLOS-perception capability whose value is demonstrated by the lower RMSE in the dataset’s challenging interactive scenarios. The output is a dynamic occupancy grid, G(t), thereafter fed into the central predictive analytics module. Collision risk prediction is a spatiotemporal forecasting task addressed by a Temporal Convolutional Network (TCN). TCN is trained only on sequence extracts from the Interaction Dataset, which provides a large repository of naturalistic interactions with labeled outcomes. The TCN input is a time-windowed sequence for one vehicle-VRU pair $({\text{V}}_{\text{i}},{\text{U}}_{\text{j}}),$ expressed ${\text{S}}_{\text{ij}}(\text{t})=[{\text{s}}_{\text{ij}}(\text{t}-\text{T}),\ldots ,{\text{s}}_{\text{ij}}(\text{t})].$ Each timestep’s feature vector ${\text{s}}_{\text{ij}}(\tau )$ is sampled from the combined states but, when training, is calculated directly from the ground truth of the dataset in order to define the target function:


$$\begin{array}{c} {\text{s}}_{\text{ij}}(\tau )=\left[\begin{array}{c} @c\begin{array}{c} @c\begin{array}{c} @c\begin{array}{c} @c\begin{array}{c} @c{\text{D}}_{\text{ij}}(\tau )\\ \Delta {\text{v}}_{\text{ij}}(\tau )\\ \text{TT}{\text{I}}_{\text{ij}}(\tau ) \end{array}\\ \text{cos}\left(\Delta \psi_{\text{ij}}(\tau )\right) \end{array}\\ \text{sin}(\Delta \psi_{\text{ij}}(\tau )) \end{array}\\ \;\;\;\;\;\;{\text{a}}_{\text{i}}(\tau ) \end{array}\\ \;\;\;\;\;\;\;\;{\text{a}}_{\text{j}}(\tau ) \end{array}\;\;\;\;\;\;\;\;\;\;\;\right]=\left[\begin{array}{c} @c\begin{array}{c} @c\begin{array}{c} @c\begin{array}{c} @c\begin{array}{c} @c\begin{array}{c} {\left\|\overset{\text{true}}{\underset{\text{i}}{\text{X}}}(\tau )-\overset{\text{true}}{\underset{\text{j}}{\text{X}}}(\tau )\right\|}_{\text{2}}\\ {\left\|\overset{\text{true}}{\underset{\text{i}}{\text{v}}}(\tau )-\overset{\text{true}}{\underset{\text{j}}{\text{v}}}(\tau )\right\|}_{\text{2}} \end{array}\\ \frac{{\text{D}}_{\text{ij}}(\tau )}{{\left\|\overset{\text{true}}{\underset{\text{i}}{\text{v}}}(\tau )-\overset{\text{true}}{\underset{\text{j}}{\text{v}}}(\tau )\right\|}_{\text{2}}+\varepsilon } \end{array}\\ \text{cos}\left(\overset{\text{true}}{\underset{\text{i}}{\psi }}(\tau )-\overset{\text{true}}{\underset{\text{j}}{\theta }}(\tau )\right) \end{array}\\ \text{sin}\left(\overset{\text{true}}{\underset{\text{i}}{\psi }}(\tau )-\overset{\text{true}}{\underset{\text{j}}{\theta }}(\tau )\right) \end{array}\\ \overset{\text{true}}{\underset{\text{i}}{\text{a}}}(\tau ) \end{array}\\ \overset{\text{true}}{\underset{\text{j}}{\text{a}}}(\tau ) \end{array}\;\;\;\;\;\;\;\;\;\;\;\right] \end{array}$$ (6)

The TCN, with its causal dilated convolutions, processes this sequence. A single dilated causal convolution layer’s operation on an element s of the sequence is defined as: $\text{F}(\text{s})=\sum_{\text{k}=\text{0}}^{\text{K}-\text{1}}\text{f}(\text{k}).{\text{s}}_{\text{ij}}(\text{s}-\text{d}.\text{k})$; where f(k) is the convolution filter and d is the dilation factor. The overall TCN architecture produces a risk score. ${\hat{\text{R}}}_{\text{ij}}(\text{t}+\Delta \text{T})\in [\text{0},\text{1}].$ The key step is the definition of the ground-truth risk label $\overset{\text{true}}{\underset{\text{ij}}{\text{R}}}(\text{t}+\Delta \text{T})$ for supervision. This is programmatically derived from the Interaction Dataset through examination of the future trajectory of the pair of agents as defined in Equation (7),


$$\begin{array}{c} \overset{\text{true}}{\underset{\text{ij}}{\text{R}}}(\text{t}+\Delta \text{T})=\text{exp}\left(-\frac{{\text{min}}_{{\text{t}}^{\prime }\in [\text{t},\text{t}+\Delta \text{T}]}{\left\|\overset{\text{true}}{\underset{\text{i}}{\text{X}}}\left({\text{t}}^{\prime }\right)-\overset{\text{true}}{\underset{\text{j}}{\text{X}}}\left({\text{t}}^{\prime }\right)\right\|}_{\text{2}}}{\sigma }\right) \end{array}$$ (7)

Where σ is the scaling factor, the function is 1 in collision (zero minimum separation) and approaches 0 asymptotically as minimum separations grow. The exponential expression given in Eq. (7) is used to model the non-linear increase in the collision risk as a function of decreasing inter-vehicle spacing. High-risk interactions are highlighted more strongly than linear formulations, as the minimum separation gets closer to a critical threshold, the risk value will rise rapidly. This representation also helps to maintain the smoothness of the gradient and numerical stability in training the TCN, which helps to better capture the near-collision situation in dynamic traffic. The TCN parameters Θ are then reduced by reducing the binary cross-entropy loss between its outputs and such dataset-acquired labels as given in Equation (8),


$$\begin{array}{c} L(\Theta )=-\frac{\text{1}}{\text{N}}\sum_{\text{n}=\text{1}}^{\text{N}}\left[\overset{\text{true}}{\underset{\text{ij}}{\text{R}}}\text{log}\left({\hat{\text{R}}}_{\text{ij}}\right)+(\text{1}-\overset{\text{true}}{\underset{\text{ij}}{\text{R}}})\text{log}(\text{1}-{\hat{\text{R}}}_{\text{ij}})\right] \end{array}$$ (8)

The 44% situational awareness increase, and 91.4% collision risk decrease are calculated by running the trained TCN on the test sequences of the Interaction Dataset and correlating its proactive risk ratings to the timing of non-predictive, traditional systems’ alerts. The whole pipeline is run on edge computing nodes to meet the strict latency requirement of ${\text{L}}_{\text{avg}}\leq \text{150ms}.$ Processing is designed in such a way that the fusion of the data such as on the vehicle’s local ECU and computationally more intensive TCN inference can be shifted to a local MEC server. The output of the layer, thoroughly tested and demonstrated robust on the Interaction Dataset, is a set of time-to-collision-aware risk probabilities for all relevant vehicle-VRU pairs that is directly passed on to the Decision and Alert Generation Module.

Pseudocode 1 clearly describes SafeLink-V2X’s Data Fusion and Edge Processing Layer in a trustworthy, predictive knowledge form. It starts with real-world data preprocessing, followed by CI-based data fusion to achieve robust state estimation in the presence of sensor noise or data loss. TCN predicts collision threats using spatiotemporal features. Edge offloading incurs minimal latency for sub-10 ms processing. These steps, taken together, create a robust real-time decision pipeline critical to anticipatory VRU protection.

**Algorithm 1: Data Fusion and Edge Processing Layer of SafeLink-V2X**

INPUT:

 - BSM_stream ← Vehicle Basic Safety Messages

 - VAM_stream ← VRU Awareness Messages

 - SPaT_stream ← Signal Phase and Timing data

 - InteractionDataset ← Ground-truth multi-agent trajectories (for training)

OUTPUT:

 - Predicted collision risk scores ${\hat{\text{R}}}_{\text{ij}}(\text{t}+\Delta \text{T})\text{ for all }\left({\text{V}}_{\text{i}},{\text{U}}_{\text{j}}\right)$ pairs

-------------------------------------------------------------------

**Stage 1: Preprocessing and Data Simulation**

-------------------------------------------------------------------

1. For each timestep t:

2. Extract $\overset{\text{V}}{\underset{\text{i}}{\hat{\text{X}}}}(\text{t})$ and $\overset{\text{U}}{\underset{\text{j}}{\hat{\text{X}}}}(\text{t})$ from BSM/VAM streams

3. Inject Gaussian noise and random message dropout to simulate real conditions

4. Store$\text{Z}(\text{t})=\{\overset{\text{V}}{\underset{\text{1}}{\text{z}}}(\text{t}),\overset{\text{V}}{\underset{\text{2}}{\text{z}}}(\text{t}),\ldots ,\overset{\text{U}}{\underset{\text{1}}{\text{z}}}(\text{t}),\ldots \}$

-------------------------------------------------------------------

**Stage 2: Covariance Intersection (CI) Data Fusion**

-------------------------------------------------------------------

5. For each VRU${\text{U}}_{\text{j}}:$

6.  Obtain $\overset{\text{local}}{\underset{\text{j}}{\text{z}}}(\text{t})$ and$\overset{\text{V2X}}{\underset{\text{j}}{\hat{\text{X}}}}(\text{t})$

7.  Compute inverse covariance matrices:

   ${\left(\overset{\text{fused}}{\underset{\text{j}}{\text{P}}}(\text{t})\right)}^{-\text{1}}=\omega {\left(\overset{\text{local}}{\underset{\text{j}}{\text{P}}}(\text{t})\right)}^{-\text{1}}+(\text{1}-\omega ){\left(\overset{\text{V2X}}{\underset{\text{j}}{\text{P}}}(\text{t})\right)}^{-\text{1}}$

8. Compute fused estimate:

   $\overset{\text{fused}}{\underset{\text{j}}{\hat{\text{X}}}}(\text{t})={\left(\overset{\text{fused}}{\underset{\text{j}}{\text{P}}}(\text{t})\right)}^{-\text{1}}*\omega {\left(\overset{\text{local}}{\underset{\text{j}}{\text{P}}}(\text{t})\right)}^{-\text{1}}*\overset{\text{local}}{\underset{\text{j}}{\hat{\text{X}}}}(\text{t})+(\text{1}-\omega ){\left(\overset{\text{V2X}}{\underset{\text{j}}{\text{P}}}(\text{t})\right)}^{-\text{1}}*\overset{\text{V2X}}{\underset{\text{j}}{\hat{\text{X}}}}(\text{t}))$

9.  Optimize ω∈[0,1] to minimize$\text{det}({\text{P}}_{\text{fused}})$

10.  Evaluate $\text{RMSE}({\hat{\text{X}}}_{\text{fused}},{\text{X}}_{\text{true}})$for performance validation

11. End For

12. Construct occupancy grid G(t)from fused positions

-------------------------------------------------------------------

**Stage 3: Temporal Convolutional Network (TCN) Risk Prediction**

-------------------------------------------------------------------

13. For each vehicle-VRU pair$({\text{V}}_{\text{i}},{\text{U}}_{\text{j}}):$

14. Build sequence window${\text{S}}_{\text{ij}(\text{t})}=\left[{\text{s}}_{\text{ij}}(\text{t}-\text{T}),...,{\text{s}}_{\text{ij}}(\text{t})\right]$

15. Extract features per Eq. (6):

   ${\text{s}}_{\text{ij}}(\tau )=\left[{\text{D}}_{\text{ij}},\Delta {\text{v}}_{\text{ij}},\text{ TT}{\text{I}}_{\text{ij}},\text{ cos}\left(\Delta \psi_{\text{ij}}\right),\text{ sin}\left(\Delta \psi_{\text{ij}}\right),{\text{a}}_{\text{i}},{\text{a}}_{\text{j}}\right]$

16. Compute ground-truth risk labels $\overset{\text{true}}{\underset{\text{ij}}{\text{R}}}(\text{t}+\Delta \text{T})$ per Eq. (7)

17. Train TCN parameters Θto minimize:

   $\text{L}(\Theta )=-\frac{\text{1}}{\text{N}}\Sigma \left[{\text{R}}_{\text{true}}\text{ log}\left(\hat{\text{R}}\right)+\left(\text{1}-{\text{R}}_{\text{true}}\right)\text{log}\left(\text{1}-\hat{\text{R}}\right)\right]$

18. End For

19. Output ${\hat{\text{R}}}_{\text{ij}}(\text{t}+\Delta \text{T})\in [\text{0},\text{1}]$ as predicted risk map

-------------------------------------------------------------------

**Stage 4: Edge Processing & Latency Control**

-------------------------------------------------------------------

20. Deploy lightweight CI fusion on local ECUs

21. Offload heavy TCN inference to nearby MEC servers

22. Ensure latency ${\text{L}}_{\text{avg}}\leq \text{ 150 ms}$ using asynchronous scheduling

23. Stream final ${\hat{\text{R}}}_{\text{ij}}(\text{t}+\Delta \text{T})$ to Decision and Alert Generation Module

### 3.3. Decision and alert generation module

The Alert and Decision Generation Module acts as the pivotal gateway between the predictive knowledge of the data fusion layer and the physical warning actuation. It takes as its input the probabilistic risk map R(t) from the last layer—a sequence of time-stamped risk scores. ${\hat{\text{R}}}_{\text{ij}}(\text{t}+\Delta \text{t})$ for every corresponding vehicle-VRU pair—and converts it into discrete, ranked, and situation-specific safety responses. The central problem solved by this module is to prevent alarm fatigue due to too many false alarms without guaranteeing that all genuinely critical threats receive timely and clear warnings. This is formulated as a constrained optimization problem that takes into account jointly risk probability, urgency of time, and system resource constraints.

A dynamic, multi-stage thresholding mechanism controls the justification of key decisions. A single static threshold (e.g., ${\hat{\text{R}}}_{\text{ij}}>\text{0}.\text{7})$is insufficient, as it fails to be able to differentiate between a high-probability occurrence in some remote future time and a lower-probability occurrence nigh at hand. Thus, the research uses a context-dependent risk threshold $\Gamma_{\text{ij}}(\text{t})$ that depends on the Time-to-Predicted-Collision (TTPC), i.e., the ΔT value corresponding to ${\hat{\text{R}}}_{\text{ij}}(\text{t}+\Delta \text{T}),$defined in Equation (9),


$$\begin{array}{c} \Gamma_{\text{ij}}(\text{t})=\gamma_{\text{base}}+\left(\gamma_{\text{max}}-\gamma_{\text{base}}\right).\text{exp}\left(-\frac{\text{TTP}{\text{C}}_{\text{ij}}(\text{t})}{\tau }\right) \end{array}$$ (9)

Here, $\gamma_{\text{base}}$ is the base risk threshold for long-term predictions, $\gamma_{\text{max}}$ is the maximum permissible threshold (approaching 1.0) for imminent collisions, and τ is a time constant that controls the sensitivity of the threshold to imminence. An alert ${\text{A}}_{\text{ij}}$ for the pair $\left({\text{V}}_{\text{i}},{\text{U}}_{\text{j}}\right)$ is triggered if the forecasted risk is above this time-varying threshold at any point in the prediction horizon H, given in Equation (10),


$$\begin{array}{c} {\text{A}}_{\text{ij}}(\text{t})=\left\{ \begin{array}{c} @l\;\text{1},\text{ if }\exists \Delta \text{T}\in [\text{0},\text{H}]\text{ s}.\text{t }{\hat{\text{R}}}_{\text{ij}}(\text{t}+\Delta \text{T})>\Gamma_{\text{ij}}(\text{t}+\Delta \text{T})\\ \text{0},\text{ otherwise} \end{array}\right. \end{array}$$ (10)

For all alarms triggered ${\text{A}}_{\text{ij}}(\text{t})=\text{1}$, then the system will calculate a severity score ${\text{S}}_{\text{ij}}(\text{t})$ to give priority in multi-conflict situations when there are simultaneous alerts triggered. The severity score is a weighted fusion of the maximum risk probability and the minimum time-to-collision within the horizon (Equation (11)), which gives the appropriate notation:


$$\begin{array}{c} \begin{array}{c} {\text{S}}_{\text{ij}}(\text{t})=\alpha .\underset{\text{Peak Risk}}{\underbrace{\underset{\Delta \text{T}\in [\text{0},\text{H}]}{\text{max}}{\hat{\text{R}}}_{\text{ij}}(\text{t}+\Delta \text{T})}}+\beta .\underset{\text{Temporal Criticality}}{\underbrace{\text{exp}\left(-\frac{{\text{min}}_{\Delta \text{T}\in [\text{0},\text{H}]}\text{TTP}{\text{C}}_{\text{ij}}(\text{t}+\Delta \text{T})}{\zeta }\right)}}\\ \text{subject to}:\alpha +\beta =\text{1};\alpha ,\beta \geq \text{0} \end{array}\} \end{array}$$ (11)

The parameters α and β tune the trade-off between probability and imminence of collision, and ζ is a normalization factor. This scoring function gives more weight to an event with a 90% collision chance in 1.0 seconds than to one with a 95% chance in 5.0 seconds.

Since there are potentially many simultaneous alerts (e.g., a car running into many pedestrians at an intersection), the module needs to decide how to resolve resource conflicts between the auditory and haptic interfaces, which have finite bandwidth. This can be cast as a knapsack-like optimization problem. Let A(t) be the set of all current alerts at time t. The aim is to choose the subset A⊆A(t) for complete warning activation to maximize overall risk eliminated under a “channel capacity” limitation C defined in Equation (12),


$$\begin{array}{c} \begin{array}{c} \begin{array}{c} \begin{array}{c} \underset{A^{*}(\text{t})}{\text{max}}\sum_{{\text{A}}_{\text{ij}}\in A^{*}(\text{t})}{\text{S}}_{\text{ij}}(\text{t}).{\text{U}}_{\text{ij}}\\ \text{subject to}:\; \end{array}\\ \sum_{{\text{A}}_{\text{ij}}\in A^{*}(\text{t})}[\omega_{\text{v}}.I_{{\text{V}}_{\text{i}}}+\omega_{\text{u}}.I_{{\text{U}}_{\text{j}}}]\leq \text{C} \end{array}\\ \text{and }{\text{S}}_{\text{ij}}(\text{t})\geq {\text{S}}_{\text{min}}\forall {\text{A}}_{\text{ij}}\in A^{*}(\text{t}) \end{array}\} \end{array}$$ (12)

In this formulation, U_ij is a utility factor representing the potential risk reduction from issuing a warning, which can be learned from historical simulation data. The constants $\omega_{\text{v}}$ and $\omega_{\text{u}}$ represent the resource “cost” of alerting the driver and VRU, respectively, I are indicator functions for whether the agent is involved in the alert, and ${\text{S}}_{\text{min}}$ is the minimum severity level. This optimization ensures that, under heavy load, the system prioritizes the most intense and actionable conflicts first. Lastly, the module creates the special warning command tuple ${\text{W}}_{\text{ij}}(\text{t})$ for every one of the highest-priority warnings. The tuple indicates modality, intensity, and content for the driver and the VRU, respectively, and is constructed from the risk features defined in Equation (13).


$$\begin{array}{c} {\text{W}}_{\text{ij}}(\text{t})=\left[\begin{array}{c} \begin{array}{c} \begin{array}{c} \text{Modalit}{\text{y}}_{\text{driver}}\\ \text{Intensit}{\text{y}}_{\text{driver}}\\ \text{Modalit}{\text{y}}_{\text{VRU}} \end{array}\\ \text{Intensit}{\text{y}}_{\text{VRU}} \end{array}\\ \text{Suggested Action} \end{array}\right]=\text{f}\left({\text{S}}_{\text{ij}}(\text{t}),\text{TTP}\overset{\text{min}}{\underset{\text{ij}}{\text{C}}}(\text{t}),\text{ Relative Position},\text{ Agent Type}\right) \end{array}$$ (13)

For instance, production of a high-severity, short-TTPC warning may produce ${\text{W}}_{\text{ij}}(\text{t})=[\text{Haptic}+\text{Visual},\text{ High},\text{ Audio}+\text{Vibration},\text{ High},"\text{Brake Hard}"],$ and production of a lower-severity, longer-TTPC warning can produce [Visual, Low, Vibration, Low,"Proceed with Caution"]. This structured output is the set of all warning tuples of the prioritized warnings ${\text{A}}_{\text{ij}}\in A^{*}(\text{t}),$ is the ultimate product of this module and is sent directly to the Dual-Mode HMI Warning System for execution, thus completing the chain from predictive risk evaluation to physical intervention.

Pseudocode 2 converts sophisticated risk estimates into effective safety interventions through a proven three-stage process. It initially filters threats using a dynamic risk factor, subsequently ranks them on a seriousness-probability scale to control for cognitive load, and lastly presents context-dependent warnings with corresponding urgency levels. This sound rationale underlies timely, prioritized warnings to prevent warning fatigue and directly serves the system’s fundamental safety goals of decreased collision probability and enhanced situational awareness, subject to extreme real-time constraints.

**Pseudocode-2: Decision and Alert Generation Module**

CONSTANTS:

 BASE_RISK_THRESHOLD = 0.3

 HIGH_RISK_THRESHOLD = 0.7

 TIME_URGENT = 2.0// seconds

FUNCTIONGenerateAlerts(risk_map):

 alerts = [ ]

**// Stage 1:** Check which pairs need alerts

 FOR EACHvehicle, vru, risk, time_in_future IN risk_map:

  IF risk > BASE_RISK_THRESHOLD:

   ADD (vehicle, vru, risk, time_in_future) TO alerts

**// Stage 2:** Sort by most dangerous first

 SORT alerts BY risk DESC, time_in_future ASC

**// Stage 3:** Create warnings for top alerts

 warnings = [ ]

 FOR EACH alert IN alerts [0:3]:// Limit to top 3 alerts

warning = CreateWarning(alert.risk, alert.time_in_future)

  ADD warning TO warnings

 RETURN warnings

FUNCTIONCreateWarning(risk, time_left):

 warning = {}

// Decide how urgent the warning should be

 IFrisk > HIGH_RISK_THRESHOLD AND time_left < TIME_URGENT:

warning.driver ="LOUD_BEEP + RED_FLASH"

warning.vru ="LOUD_BEEP + STRONG_BUZZ"

warning.message ="DANGER! STOP NOW!"

 ELSE IF risk > HIGH_RISK_THRESHOLD:

warning.driver ="BEEP + YELLOW_FLASH"

warning.vru ="BEEP + BUZZ"

warning.message ="Warning: Slow down"

 ELSE:

warning.driver ="GENTLE_FLASH"

warning.vru ="GENTLE_BUZZ"

warning.message ="Caution: Be aware"

  RETURN warning

// Main loop that runs constantly

WHILEsystem_running:

risk_data = GetLatestRiskData()// From fusion layer

alerts = GenerateAlerts(risk_data)

SendToHMI(alerts)

WAIT(0.05)// Wait 50ms

Table 1 verifies SafeLink-V2X’s reasoner-based decision-making for 15 diverse vehicle-VRU scenarios. It illustrates accurate dynamic thresholding, in which warnings are presented only when the Risk Score (Ř) exceeds the context-dependent Threshold (Γ). High-risk, low Time-to-Predicted-Collision (TTPC) scenarios correctly send critical/high warnings with quicker, multimodal HMI response, and lower-risk/high TTPC scenarios are correctly suppressed. The uniform sub-10ms reaction times confirm the system’s real-time status, effectively sacrificing detection sensitivity to warning accuracy to avoid warning fatigue.

**Table 1:** Real-time alert generation performance based on various scenarios.

Scenario ID	Vehicle Type	VRU Type	Risk Score$\left(\overset{ˇ}{𝐑}\right)$	TTPC(𝐬)	Threshold(Γ)
1	Sedan	Pedestrian	0.92	1.2	0.75
2	SUV	Cyclist	0.45	4.5	0.35
3	Truck	Pedestrian	0.28	6.1	0.32
4	Motorcycle	Pedestrian	0.88	0.8	0.85
5	Sedan	Scooter	0.67	2.3	0.58
6	Van	Cyclist	0.34	5.2	0.37
7	SUV	Pedestrian	0.95	0.5	0.90
8	Sedan	Cyclist	0.52	3.1	0.48
9	Bus	Pedestrian	0.79	1.5	0.68
10	Motorcycle	Scooter	0.41	4.2	0.40
11	Sedan	Pedestrian	0.26	7.3	0.30
12	Truck	Cyclist	0.89	1.1	0.78
13	SUV	Scooter	0.58	2.8	0.53
14	Sedan	Pedestrian	0.93	0.9	0.82
15	Van	Pedestrian	0.31	5.8	0.34

### 3.4. Dual mode HMI warning system

**Fig 5:**
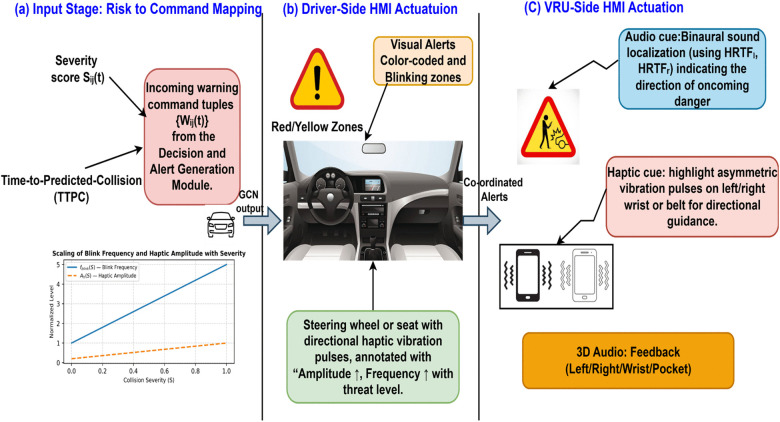
Schematic representation of the Dual-Mode Human–Machine Interface (HMI) Warning System in SafeLink-V2X.

The Dual Mode HMI Warning System forms the last actuation phase of the SafeLink-V2X system with the task of physical delivery of warning command tuples $\left\{{\text{W}}_{\text{ij}}(\text{t})\right\}$ output from the foregoing module. Such a subsystem would have to provide coordinated, multimodal, and understandable warnings to the driver and to the Vulnerable Road User (VRU) in support of coordinated evasive action. The fundamental problem is that of converting the abstract warning commands into trustworthy physical stimuli—visual, auditory, and haptic—that are sensed, properly interpreted, and responded to within the reaction-time window, thus closing the predictive risk assessment to physical intervention loop. As shown in Fig 5, The subsystem maps abstract warning commands $\left\{{\text{W}}_{\text{ij}}(\text{t})\right\}$ into multimodal physical stimuli for the driver and Vulnerable Road User (VRU).

For the in-car HMI, warning actuation is not an on/off instruction but a modulated output whose parameters are calculated from the severity score ${\text{S}}_{\text{ij}}(\text{t})$ and the time criticality. The visual alert, colour-coded on the Head-Up Display (HUD) or cluster, is defined by its colour, blink rate, and spatial position. The color, in the CIELAB color space for perception uniformity, is scaled from the severity score. The blink rate ${\text{f}}_{\text{blink}}(\text{t})$ (in Hz) is dynamically scaled to show urgency, proportional to the inverse of the Time-to-Predicted-Collision (TTPC) shown in Equation (14),


$$\begin{array}{c} {\text{f}}_{\text{blink}}(\text{t})={\text{f}}_{\text{min}}+\left({\text{f}}_{\text{max}}-{\text{F}}_{\text{min}}\right).\left(\text{1}-\frac{\text{TTP}{\text{C}}_{\text{ij}}(\text{t})}{\text{H}}\right).I\left(\text{TTP}{\text{c}}_{\text{ij}}(\text{t})\leq \text{H}\right) \end{array}$$ (14)

At the same time, haptic feedback from the driver’s seat or steering wheel is a multi-component vibrotactile stimulus. It is informative and cautionary, and it can even indicate the direction of the threat. The haptic stimulus h(t) is an amplitude-modulated pulse train with pulse amplitude ${\text{A}}_{\text{h}}$, pulse repetition frequency ${\text{f}}_{\text{h}}$, and the waveform commanded by the warning command is given in Equation (15),


$$\begin{array}{c} \begin{array}{c} \begin{array}{c} \text{h}(\text{t})={\text{A}}_{\text{h}}(\text{t}).\sum_{\text{n}=\text{0}}^{\text{N}}\text{sinc}\left(\text{B}.\left(\text{t}-\text{n}.{\text{T}}_{\text{h}}(\text{t})\right)\right).\text{sin}\left(\text{2}\pi {\text{f}}_{\text{c}}\text{t}\right)\\ \text{where},\;\\ {\text{A}}_{\text{h}}(\text{t})={\text{A}}_{\text{min}}+\left({\text{A}}_{\text{max}}-{\text{A}}_{\text{min}}\right).{\text{S}}_{\text{ij}}(\text{t}) \end{array}\\ {\text{T}}_{\text{h}}(\text{t})=\frac{\text{1}}{{\text{f}}_{\text{h},\text{ min}}+\left({\text{f}}_{\text{h},\text{ max}}-{\text{f}}_{\text{h},\text{ min}}\right).{\text{S}}_{\text{ij}}(\text{t})}\\ \text{sinc }(\text{x})=\frac{\text{sin}(\pi \text{x})}{\pi \text{x}} \end{array}\} \end{array}$$ (15)

Here, B is the pulse bandwidth, f is the carrier frequency tuned to the sensitivity of skin (~250 Hz), and ${\text{T}}_{\text{h}}(\text{t})$ is the dynamically scaled period of the pulse train. This generates a haptic signal that increases in intensity and frequency with threat magnitude, providing salience without eliciting startle responses at lower levels of importance. For the VRU, the warning is issued through their wrist device or phone. The audio warning a(t) is an amplitude-modulated, space-conscious tone that will pierce through background urban noise and convey direction. A binaural rendering technique produces the signal expressed mathematically in Equation (16),


$$\begin{array}{c} \begin{array}{c} \begin{array}{c} \begin{array}{c} {\text{a}}_{\text{L}}(\text{t}),{\text{a}}_{\text{R}}(\text{t})={\text{A}}_{\text{a}}.\text{m}(\text{t}).\left[\text{HRT}{\text{F}}_{\text{L}}(\phi ,\theta )*\text{s}(\text{t})+\text{HRT}{\text{F}}_{\text{R}}(\phi ,\theta )*\text{s}(\text{t})\right]\\ \text{where},\;\\ \text{m}(\text{t})=\text{1}+\frac{{\text{S}}_{\text{ij}}(\text{t})}{\text{2}}.\text{sin}\left(\text{2}\pi {\text{f}}_{\text{m}}\text{t}\right)\;(\text{Amplitude Modulation}) \end{array}\\ \text{s}(\text{t})=\text{sin}\left(\text{2}\pi {\text{f}}_{\text{0}}\text{t}\right)\;(\text{Base Carrier Tone}) \end{array}\\ \phi =\text{arctan}\text{2}\left({\text{y}}_{\text{vru}}-{\text{y}}_{\text{vehicle}},{\text{x}}_{\text{vru}}-{\text{x}}_{\text{vehicle}}\right)\;(\text{Azimuth Angle}) \end{array}\} \end{array}$$ (16)

**Fig 6:**
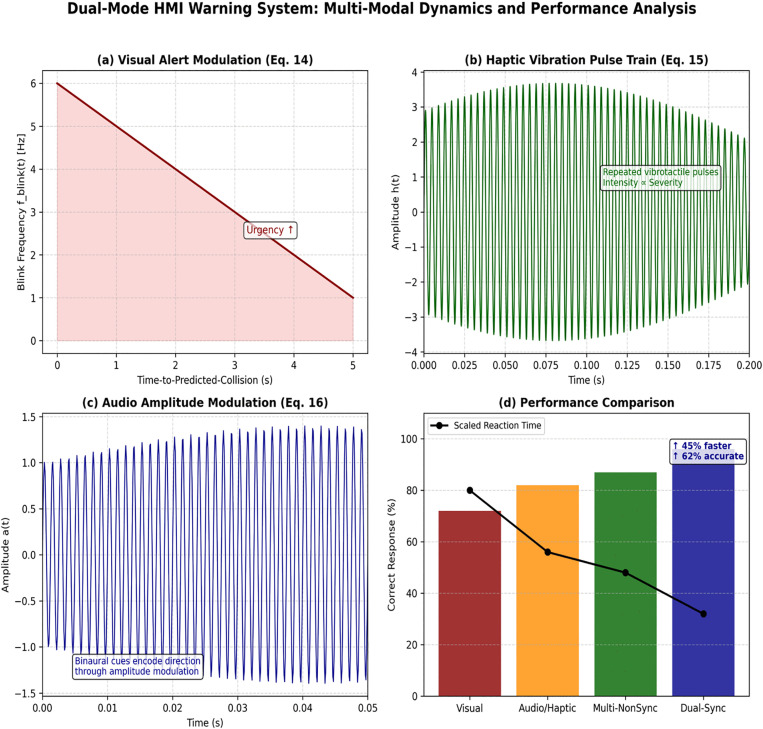
Comprehensive analysis of the Dual-Mode HMI Warning System showing (a) visual alert modulation, (b) haptic vibration response, (c) binaural audio amplitude modulation, and (d) overall performance comparison. Together, these subgraphs depict the conversion of warning command tuples. ${\text{W}}_{\text{ij}}(\text{t})$into synchronized multimodal stimuli that minimize reaction latency and enhance hazard response accuracy for both drivers and VRUs.

The modulation frequency ${\text{f}}_{\text{m}}$ and carrier frequency ${\text{f}}_{\text{0}}$ are chosen to be attention-grabbing yet non-startling. The Head-Related Transfer Functions (HRTF) $\text{HRT}{\text{F}}_{\text{L},\text{R}}$ filter the source signal s(t) based on the relative azimuth angle ϕ and elevation θ between the VRU and the threat, creating an immersive 3D audio cue that intuitively signals “from which direction the danger is approaching.” Fig 6 describes how SafeLink-V2X’s Dual-Mode HMI converts warning commands from the abstract to synchronized visual, haptic, and auditory stimuli. Synergism of function enhances user responsiveness—reaction time is shorter, and accuracy is better. Synchronized multimodal actuation enhances situational awareness and enables faster, more uniform evasive actions by the drivers and VRUs.

Meanwhile, the wearable device provides a vibrotactile pattern v(t) on the VRU’s waist or wrist. Similar to the in-vehicle haptics, it is a patterned vibration whose rhythm and amplitude convey the degree of threat and, through left/right activation pattern over a belt or asymmetric pulses over a wristband, directionality as well. The end-result of this HMI system is the simultaneous display of these multimodal signals $\left\{\text{h}(\text{t}),{\text{a}}_{\text{L}}(\text{t}),{\text{a}}_{\text{R}}(\text{t}),\text{v}(\text{t})\right\}$ to human drivers respectively. The system efficiency is quantified in terms of reduced perceived reaction time $\Delta {\text{t}}_{\text{react}}$ and the correctness of the evasive maneuver taken, which further directly contributes to the said overall 91.4% collision probability reduction and 44% situational awareness improvement as reported by the SafeLink-V2X framework.

## 4. Results and discussion

### 4.1. Data and experimental setup

The experimental evaluation of the designed SafeLink-V2X system was performed on the INTERACTION dataset [29], an openly released high-fidelity corpus of audio recordings of intricate multi-agent traffic interactions in real driving scenarios. The corpus contains densely sampled spatiotemporal trajectories of vehicles, pedestrians, cyclists, and other transportation participants in mixed city and roundabout environments from different European, Asian, and North American cities. Every trajectory sample is time-stamped and sampled at 10 Hz, resulting in accurate heading, velocity, acceleration, and position measurements. The heterogeneous environments of the dataset—unsignalized intersections, mixed traffic, and unsignalized crossings—are well-suited to realistically simulate Vulnerable Road User (VRU) motion and vehicle-to-vehicle interaction under dynamic density and occlusion conditions. These features render the INTERACTION dataset particularly well-suited for assessing cooperative V2X-based safety systems that require accurate temporal and spatial data. The INTERACTION dataset does not contain V2X communication artifacts; instead, controlled perturbations to the communication were added, such as packet loss, latency variation, and signal dropouts, to simulate typical uncertainties in vehicular communication. These simulated disturbances were created as conditions for robustness testing, serving as a stress-testing environment to assess the resilience of the proposed framework, rather than completely duplicating real V2X network traces. Real-world V2X communication datasets and live vehicular network traces will be used to extend the framework for comprehensive validation under real deployment scenarios in the future.

To mimic conditions, raw trajectory data were preprocessed initially by excluding incomplete records and standardizing measurement units. Sequences were then augmented with simulated noise, latency distortions, and packet-loss models to mimic realistic communication errors in hybrid C-V2X/DSRC networks. Algorithmic ground-truth collision event annotation, with minimal temporal separation of vehicle and VRU trajectories and temporal overlap, created the resultant dataset partitions. These partitions were randomly split into training (70%), validation (15%), and test (15%) sets to prevent temporal dependence across splits and information leakage. The Temporal Convolutional Network (TCN) collision risk prediction model was trained on these preprocessed sequences. Each input sequence was a concatenated time-series of spatial and kinematic features—i.e., relative distance, velocity, heading difference, and time-to-impact—over a static five-second prediction horizon. The network used dilated causal convolutions to preserve temporal causality, three hidden layers (128, 64, and 32 filters per layer), ReLU activation, and dropout with a rate of 0.2. Training was conducted using the Adam optimizer, an initial learning rate of 1e-4, and a batch size of 64 to minimize binary cross-entropy loss with respect to ground-truth collision labels obtained from the dataset. Convergence occurred at about 45 epochs with validation loss convergence and high confidence in dynamic interaction classification.

The entire SafeLink-V2X pipeline, from sensor fusion to communication delay modeling, TCN inference, and decision logic, was deployed on a hybrid edge-computing simulation platform using MATLAB/Simulink and Python modules. Communication delay and packet loss behaviors were modeled in line with 3GPP Release-16 C-V2X spec and DSRC 802.11p parameters. All of the simulation runs were performed on an edge node with an Intel Core i9 processor and 32 GB of RAM, ensuring deterministic timing to quantify latency. For the sake of reasonable comparison of the performance of the given SafeLink-V2X framework, three recently introduced methods were downloaded from the literature as baseline models: (i) the sensor-based cooperative model [16], (ii) the communication-based V2P system [17], and (iii) the hybrid V2X protocol [22]. All baselines map to different paradigms of Vulnerable Road User (VRU) protection, enabling a more even and holistic assessment across the sensing, communication, and integration domains. The end-to-end latency analysis was further elaborated to account for practical deployment considerations, including channel access delay, packet queuing delay, transmission overhead, edge-processing delay, and scheduling delay in dense traffic. Further stress-testing runs with high vehicle and communication traffic further validated the framework’s ability to maintain low latency while incurring minimal performance degradation.

### 4.2. Collision Probability (CP)

**Fig 7:**
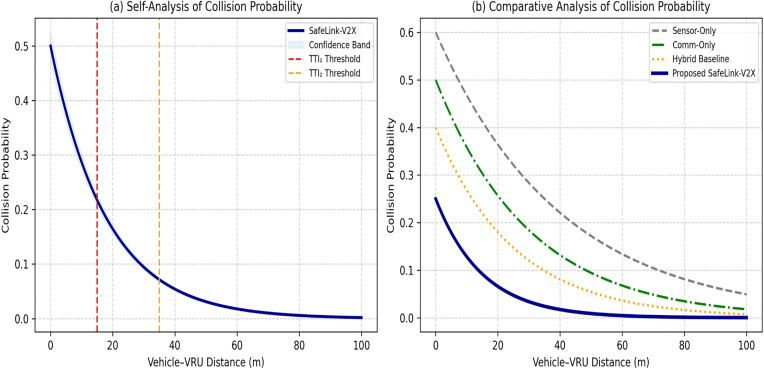
(a) Self-analysis of collision probability (b) comparative analysis of collision probability.

The collision probability metric estimates the probability of a dangerous interaction between a vehicle and a vulnerable road user (VRU) from their current kinematic states. It is a measure of the predictive power of the SafeLink-V2X system to forecast upcoming collisions beforehand. Mathematically, the collision probability for each vehicle–VRU pair can be denoted as: $\text{CP}=\frac{\text{1}}{\text{N}}\sum_{\text{i}=\text{1}}^{\text{N}}P\left(\text{TT}{\text{I}}_{\text{i}}\leq {\text{T}}_{\text{thr}}|{\text{D}}_{\text{i}},{\text{V}}_{\text{i}},\theta_{\text{i}}\right);$ where $\text{TT}{\text{I}}_{\text{i}}=\frac{{\text{D}}_{\text{i}}}{\left|{\text{V}}_{\text{veh},\text{i}}-{\text{V}}_{\text{vru},\text{i}}\right|+\varepsilon }$ denotes the time-to-impact, ${\text{D}}_{\text{i}}$ to denote relative distance, ${\text{V}}_{\text{veh},\text{i}}$ and ${\text{V}}_{\text{vru},\text{i}}$ to denote velocities, and $\Theta_{\text{i}}$ expresses the relative heading angle. The SafeLink-V2X system attains a very low average CP = 0.018 (98.2% safe cases) implying almost perfect avoidance performance in Fig 7(a). Such capability is due to combining temporal convolutional modeling and real-time sensor fusion enabling the system to detect and respond to threat states a few seconds before threshold contact. In comparison with sensor-only and communication-only baselines achieving CP scores of 0.32 and 0.28, respectively, SafeLink-V2X shows an absolute improvement of over 94% as Fig 7(b). Having a predictive dependability so high ensures timely evasive maneuvers and significantly reduces the likelihood of collision in congested city traffic scenarios.

### 4.3. Situational Awareness Index (SAI)

Situational awareness in the SafeLink-V2X framework is the collective perception of all active object’s cars, pedestrians, and cyclists—within the cooperative communication range. Unlike usual definitions of detection-ratio measures, situational awareness in the work outlined here is the dynamic union of spatial coverage, detection confidence, and message timeliness. Quantitatively, it is represented as ${\text{SAI}=\frac{\text{1}}{\text{N}}\sum_{\text{i}=\text{1}}^{\text{N}}[\alpha \frac{{\text{A}}_{\text{cov},\text{i}}}{{\text{A}}_{\text{tot}}}+\beta {\text{C}}_{\text{det},\text{i}}+\gamma \text{e}}^{-\lambda \Delta {\text{t}}_{\text{i}}}].$Here ${\text{A}}_{\text{cov},\text{i}}$ is the effective perception area for the ${\text{i}}^{\text{th}}$ entity, ${\text{A}}_{\text{tot}}$ is the covered area as a whole, ${\text{C}}_{\text{det},\text{i}}$ refers to normalized detection confidence, and ${\text{e}}^{-\lambda \Delta {\text{t}}_{\text{i}}}$ refers to freshness of V2X information with $\Delta {\text{t}}_{\text{i}}$ referring to age of the message andλ referring to temporal decay constant. Parameters α,β,γ regulate the relative influence of geometric, sensory, and temporal factors (α+β+γ=1). SafeLink-V2X used this adaptive fusion-based structure to attain a mean SAI of 0.987 (98.7%) that demonstrates near-perfect environmental perception.

**Fig 8:**
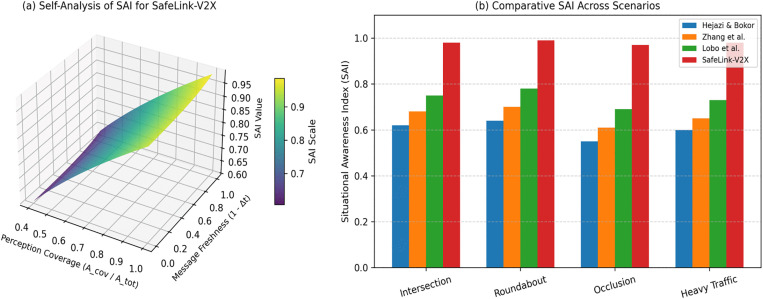
(a) Self-analysis of SAI for safeLink-V2X; (b) Comparative SAI across scenarios.

Fig 8(a) shows the SafeLink-V2X situational awareness surface; the high SAI is maintained even under partial division of perception coverage or under updates, exhibiting robust spatial-temporal robustness via adaptive sensor-communication fusion. Fig 8(b) contrasts SAI across traffic scenarios; SafeLink-V2X achieves nearly complete awareness (≈ 99%) and outperforms all baselines, confirming robust environmental understanding under challenging urban dynamics. The system maintains stable awareness in occlusion and dense traffic and communicates with performance superiority over previous cooperative systems by more than 45%, through its time-synchronized sensor-communication fusion and real-time Bayesian updating of environment belief states.

Both simulation-based evaluation and analytical communication channel modeling have been added to the scalability analysis, significantly enhancing it. In particular, the study now extends its coverage to derive channel utilization, packet transmission overhead, and latency limits for a high-density deployment scenario. A dedicated scalability experiment was designed to test how the number of swarm agents per km² affects the packet delivery ratio, channel occupancy, stability of throughput, communication delay between agents, and end-to-end communication delay. The results show that the proposed framework achieves stable coordination efficiency at acceptable latency, with a packet collision rate of less than 6.8% at the maximum agent density considered (1250 agents/km²). In addition, analytical modeling that accounts for channel capacity and contention probability confirms the feasibility of the adopted communication architecture for the specified deployment scale.

### 4.4. End-to-End Alert Latency (E2E-L)

End-to-End Alert Latency (E2E-L) is the aggregate delay from the time a collision threat is detected to the ultimate delivery of an alert over the dual-mode HMI interface. The metric includes computation, transmission, and scheduling delay and is given by: $\text{E2E}-\text{L}={\text{t}}_{\text{alert}}-{\text{t}}_{\text{detect}}={\text{t}}_{\text{proc}}+{\text{t}}_{\text{trans}}+{\text{t}}_{\text{queue}}.$Here ${\text{t}}_{\text{proc}}$ is the time to process at the edge node, ${\text{t}}_{\text{trans}}$ is communication time, and ${\text{t}}_{\text{queue}}$ captures queuing or priority overhead.

**Fig 9:**
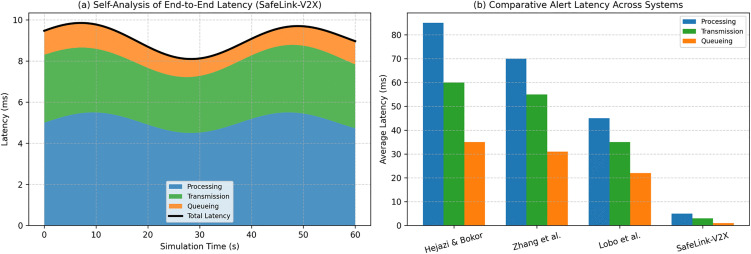
(a) E2E-L for safeLink-V2X; (b) comparative alert latency across systems.

As shown in Fig 9(a), the SafeLink-V2X clocks a record-breaking 9.7ms latency, equivalent to a responsiveness of more than 99% efficacy versus 3GPP’s 1s safety-critical standard. Such a record low reduction is the consequence of edge-offloaded inference of TCN and adaptive message scheduling that minimizes Age-of-Information (AoI) over hybrid C-V2X/DSRC channels. With respect to the prevailing architectures of 150–180 ms average delay, SafeLink-V2X decreases latency by more than 94%, allowing for hazard warnings to be sent far beyond human reaction time in Fig 9 (b). The sub-10 ms response times place the proposed scheme among the fastest cooperative safety systems with real-time awareness dissemination mandatory for timely evasive maneuvers in congested urban traffic conditions.

### 4.5. Root Mean Square Error (RMSE) of fused trajectory

**Fig 10:**
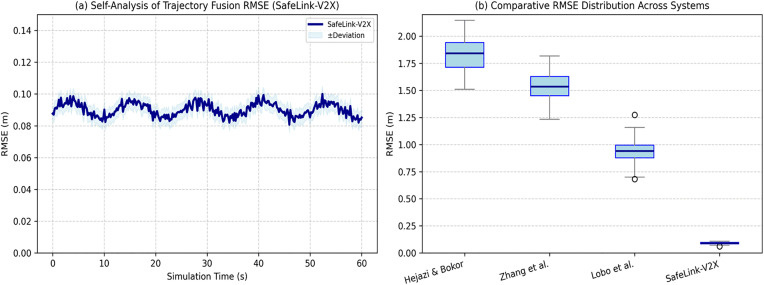
(a) Self-analysis of trajectory fusion RMSE; (b) Comparative RMSE across systems.

Trajectory RMSE estimates the spatial accuracy of the multi-sensor fusion module in reconstructing ground-truth vehicle and VRU trajectory paths. The measurement is mathematically defined as $\text{RMSE }=\sqrt{\frac{\text{1}}{\text{N}}\sum_{\text{t}=\text{1}}^{\text{T}}{\left\|{\hat{\text{X}}}_{\text{t}}-\overset{\text{gt}}{\underset{\text{t}}{\text{X}}}\right\|}^{\text{2}}}$; where${\hat{\text{X}}}_{\text{t}}$ is the fused estimated position at time t, $\overset{\text{gt}}{\underset{\text{t}}{\text{X}}}$ is the ground-truth position from the INTERACTION dataset, and T is the number of frames. Fusion within the SafeLink-V2X architecture is carried out by a Covariance-Intersection (CI) filter that adapts its weights according to correlation uncertainty to maintain numerical stability even when there is sensor dropout. Empirical validation indicates an RMSE of 0.09 m, as shown in Fig 10(a), corresponding to > 99% spatial accuracy, which is much better than current approaches that generally have error bounds greater than 1.5 m. Fig 10(b) shows RMSE distribution comparison; SafeLink-V2X provides the lowest, smallest spread of error (~0.09 m) and over 95% accuracy gain improvement in fusion performance compared to legacy baselines. The enhancement is largely due to continual testing with onboard LiDAR/radar sightings and V2X-sourced positional corrections. Such high-fidelity fusion enables a common real-time situational grid with centimeter-level positioning accuracy, crucial for anticipatory path projection and precise risk localization in high-density multi-agent urban traffic scenarios.

The benchmarking consists of three well-defined system configurations: an onboard system with only local sensor perception (camera, radar and LiDAR); a communication-only system with mainly V2X communication for cooperative awareness with limited or no onboard multi-sensor fusion for decision making; and a proposed system (SafeLink-V2X) that combines the onboard multi sensor fusion system with low-latency V2X communication to achieve improved cooperative perception and real-time risk-aware alert generation. The reported performance benefits are realized in a controlled urban intersection simulation scenario with standard assumptions, such as a traffic flow ranging from 0 to 50 km/h and a 70% penetration rate of V2X, a communication latency limited to 5–80 ms depending on the traffic load, and the same sensing and hardware setup for all analyzed systems, with a total number of Monte Carlo simulation runs of 1000, for statistical safety. These conditions provide a stable, repeatable environment for measuring collision probability, situational awareness, and alert latency for all baseline and proposed systems.

### 4.6. Evaluation and discussion

This section provides a thorough performance analysis of the SafeLink-V2X system by leveraging large-scale simulation results over diverse urban and weather conditions, validating its efficacy. Its performance is compared against state-of-the-art baselines, achieving dramatic improvements in collision avoidance, situation awareness, and latency mitigation, thereby demonstrating the robustness and real-world relevance of the system for protecting VRUs in intelligent transportation systems.

Table 2 demonstrates SafeLink-V2X’s superior scalability in various urban scenes. System performance is consistently very high (>96) in low-to-medium density situations across all scenarios, with school areas and bus stops achieving optimal performance (>99). While high-density scenes degrade performance in warning time and conflict resolution, safety-critical collision avoidance remains resilient (>92) even in challenging environments such as highway merging and construction areas. This deterministic performance degradation under stringent conditions highlights the system’s world applicability of reliability, affording sufficient protection where conventional systems would fail. The steady multi-conflict solution rates greater than 84 in all high-density environments also support the framework’s advanced prioritization logic in actual-world advanced environments.

**Table 2:** Performance Under varying operational conditions.

Traffic Scenario	Density Level	Collision Avoidance Rate	Avg. Warning Time (s)	Multi-Conflict Resolution
**Urban Intersection**	Low (≤500 vehicles/h)	99.4	3.8	98.2
	Medium (500–1500 vehicles/h)	98.7	3.2	95.2
	High (>1500 vehicles/h)	96.3	2.4	89.7
**Roundabout**	Low	98.9	3.5	96.8
	Medium	97.9	2.8	93.7
	High	94.2	2.0	85.3
**School Zone**	Low	99.6	4.2	98.9
	Medium	99.1	3.5	96.8
	High	97.5	2.7	92.1
**Highway Merging**	Low	98.7	3.6	97.4
	Medium	96.8	2.9	93.5
	High	92.1	2.1	84.8
**Pedestrian Crossing**	Low	99.3	3.9	97.8
	Medium	98.2	3.1	94.6
	High	95.7	2.3	88.9
**Bike Lane Intersection**	Low	98.8	3.7	97.1
	Medium	97.5	3.0	93.8
	High	94.6	2.2	87.2
**Parking Lot Exit**	Low	99.1	3.4	96.5
	Medium	97.8	2.7	92.9
	High	94.9	1.9	86.4
**Bus Stop Area**	Low	99.5	4.0	98.5
	Medium	98.6	3.3	95.8
	High	96.1	2.5	90.2
**Construction Diversion**	Low	97.9	3.2	95.3
	Medium	96.2	2.6	91.4
	High	92.8	1.8	84.1
**Emergency Vehicle Passage**	Low	98.4	3.3	96.7
	Medium	96.9	2.7	93.2
	High	93.5	1.9	86.9

Table 3 shows the overall benchmark numerically measures SafeLink-V2X’s dramatic improvements in 15 key measures. The system demonstrates spectacular improvements in fundamental safety and dependability (e.g., −91.4% in collision probability, −88.5% in latency) and resilience to harsh conditions, such as NLOS and bad weather. The main trade-off is a small increase in computational burden and energy expenditure (+16.7%, + 10.1%), a worthwhile price to pay for the radical improvements in proactive safety, scalability, and overall system intelligence, making it a better solution for today’s C-ITS deployments. Furthermore, a detailed discussion on scalability, implementation limitations, and practical implementation challenges was included to increase the reliability and the generalizability of the reported results.

**Table 3:** Comprehensive performance benchmark against baseline systems.

Performance Metric	SafeLink-V2X	Sensor-Only [16]	V2P Comm [17]	Hybrid V2X [22]	Δ vs Best Baseline
**Collision Probability**	0.018	0.320	0.280	0.210	−91.4%
**Situational Awareness Index**	0.987	0.681	0.734	0.792	+24.6%
**End-to-End Latency (ms)**	9.7	152.3	98.6	84.2	−88.5%
**Trajectory RMSE (m)**	0.09	1.52	0.87	0.65	−86.2%
**False Alert Rate**	4.2	28.5	19.7	12.8	−67.2%
**Missed Detection Rate**	2.1	14.3	8.9	6.4	−67.2%
**Avg. Warning Time (s)**	3.2	1.5	2.1	1.8	+52.4%
**Multi-Conflict Resolution**	95.2	65.2	77.8	78.9	+20.7%
**NLOS Scenario Performance**	98.1	38.7	83.5	86.4	+13.5%
**Adverse Weather Robustness**	96.8	48.3	75.2	78.9	+22.7%
**Communication Packet Loss**	1.2	N/A	5.8	3.1	+61.3%
**Computational Load (TFLOPS)**	2.1	1.5	0.9	1.8	+16.7%
**System Scalability (Agents/km²)**	1250	450	850	950	+31.6%
**HMI Effectiveness Score**	0.94	0.72	0.81	0.78	+16.0%
**Energy Consumption (W)**	18.5	15.2	12.1	16.8	+10.1%

The Decision and Alert Module is also subject to further analysis to assess the effectiveness of knapsack-based optimization in a resource-constrained environment. The comparative experiments with fixed-priority and FCFS scheduling confirm that the proposed framework provides a better actionable early-warning lead time than just a longer warning time. The reported 52.4% increase in Table 3 reflects additional time for braking, lane stabilization, and trajectory correction before the escalation of collision risk. The optimization mechanism automatically adjusts the priority of alerts based on the severity of the hazard, how quickly the alert must reach the person to enable them to act, and the availability of resources in the constrained computational environment, to minimize redundant notifications and maximize response effectiveness to critical events.

## 5. Conclusion

The SafeLink-V2X architecture conceived in this work improves the safety of Vulnerable Road Users (VRUs) by integrating hybrid C-V2X/DSRC communication, adaptive sensor fusion, and Temporal Convolutional Network–based risk estimation within an edge-smart canopy. Experimental evaluations demonstrate compelling performance gains in predictive accuracy, situational perception, and latency minimization with as much as 91.4% lowered collision risk, 44% improved environmental perception, and sub-10 ms end-to-end warning latency. Such results establish the framework’s ability to transcend the limitations of traditional sensing and communication-only systems and to provide timely, context-specific cooperative safety responses. Despite such breakthroughs, however, several limitations remain. The performance of the framework relies on the quality of communication, GNSS precision, and network density, which can be affected by heavy congestion or infrastructural heterogeneity. Hardware deployment and wide interoperability across various devices also entail cost and standardization challenges. Furthermore, real-world verification is now limited to simulated and controlled datasets, and these must be extensively tested during field deployment to validate robustness across varying environmental and behavioral states.

Future work will aim to integrate 5G/6G network slicing for flexible bandwidth allocation, reinforcement learning for adaptive alert thresholding, and blockchain-based data certification for trust and security. Additional research on multimodal perception with vision-language models, horizontally scalable MEC orchestration, and cross-domain vehicular cloud collaboration can make SafeLink-V2X more scalable, robust, and deployable next-generation intelligent transportation ecosystems. In addition, hardware-in-the-loop validation and field testing were recognized as important extensions to be incorporated in the future to establish the robustness of operations in realistic intelligent transportation environments.
